# The Holistic Impact of Using Arts-Based Intervention for Elderly With Dementia

**DOI:** 10.1093/geroni/igaa057.1874

**Published:** 2020-12-16

**Authors:** Rainbow Tin Hun Ho

**Affiliations:** The University of Hong Kong, Hong Kong

## Abstract

The use of creative arts on supporting elderly with dementia has been becoming popular due to its safe and engaging process. This non-pharmacological approach can complement with other treatment methods to support elderly with dementia on various aspects, including physical, cognitive and social functioning. In our randomized controlled trial on dance movement therapy (DMT) for 204 community dwelling elders with mild dementia, we found DMT could significantly reduce the level of depression, loneliness and negative mood (β=0.33-0.42, p<.01), and also the diurnal cortisol slope (β =0.30, p<.01); while in another trial on 73 elderly with moderate dementia, we found music and movement could help reduce the behavioral and psychological symptoms such as agitation (β = -0.41, p<.01), aberrant motor behavior (β=-1.02, p<.01), and dysphonia (β=-0.61, p<.05). The present presentation aims to share with the audience our practical experiences, the research procedures as well as the findings of the projects.

